# Hypoxia-Dependent Expression of TG2 Isoforms in Neuroblastoma Cells as Consequence of Different MYCN Amplification Status

**DOI:** 10.3390/ijms21041364

**Published:** 2020-02-18

**Authors:** Monica Currò, Nadia Ferlazzo, Maria Laura Giunta, Angela Simona Montalto, Tiziana Russo, Salvatore Arena, Pietro Impellizzeri, Daniela Caccamo, Carmelo Romeo, Riccardo Ientile

**Affiliations:** 1 Department of Biomedical and Dental Sciences and Morphofunctional Imaging, University of Messina, 98125 Messina, Italy; moncurro@unime.it (M.C.); nferlazzo@unime.it (N.F.); marialauragiu@hotmail.com (M.L.G.); dcaccamo@unime.it (D.C.); 2 Department of Human Pathology of Adult and Childhood “Gaetano Barresi,” University of Messina, 98125 Messina, Italy; asmontalto@libero.it (A.S.M.); russotiziana82@gmail.com (T.R.); salarena@unime.it (S.A.); impellizzerip@unime.it (P.I.); romeoc@unime.it (C.R.)

**Keywords:** HIF-1α, hypoxia, MYCN, neuroblastoma, transglutaminase isoforms

## Abstract

Transglutaminase 2 (TG2) is a multifunctional enzyme and two isoforms, TG2-L and TG2-S, exerting opposite effects in the regulation of cell death and survival, have been revealed in cancer tissues. Notably, in cancer cells a hypoxic environment may stimulate tumor growth, invasion and metastasis. Here we aimed to characterize the role of TG2 isoforms in neuroblastoma cell fate under hypoxic conditions. The mRNA levels of TG2 isoforms, hypoxia-inducible factor (HIF)-1α, *p16*, cyclin D1 and B1, as well as markers of cell proliferation/death, DNA damage, and cell cycle were examined in SH-SY5Y (non-*MYCN*-amplified) and IMR-32 (*MYCN*-amplified) neuroblastoma cells in hypoxia/reoxygenation conditions. The exposure to hypoxia induced the up-regulation of *HIF-1α* in both cell lines. Hypoxic conditions caused the up-regulation of TG2-S and the reduction of cell viability/proliferation associated with DNA damage in SH-SY5Y cells, while in IMR-32 did not produce DNA damage, and increased the levels of both TG2 isoforms and proliferation markers. Different cell response to hypoxia can be mediated by TG2 isoforms in function of *MYCN* amplification status. A better understanding of the role of TG2 isoforms in neuroblastoma may open new venues in a diagnostic and therapeutic perspective.

## 1. Introduction

Tissue transglutaminase (TG2) is a ubiquitous Ca^2+^-dependent enzyme that catalyzes transamidation/cross-linking and deamination reactions, and also acts as a protein disulfide isomerase, and a protein kinase. TG2 also has G-protein functions and several other functions based on its noncovalent interactions with different cellular proteins [1]. TG2 participates in a variety of cellular processes, including adhesion, growth, survival, apoptosis, differentiation, and extracellular matrix organization, thus contributing to a variety of physiological responses and pathological states, such as wound healing, inflammation, autoimmunity, neurodegeneration, and tissue fibrosis [1,2]. In addition, it has been described as a key enzyme in cancer cell biology involved in all stages of carcinogenesis [3]. In this context, its role is still under debate, since it has been reported to exert both pro- and anti-tumor effects [4,5]. TG2 different functions are dependent on cell types, cellular localization and specific conditions, such as calcium availability or polyamine abundance [3]. Preliminary results showed that the induction of *TGM2* gene transcription and enzyme activity is essential for the differentiating effects of retinoids in neuroblastoma cells. In particular, it has been shown that the two major TG2 protein isoforms exert opposite effects, namely the short form (TG2-S) induces neuroblastoma cell differentiation, whereas the long form (TG2-L) represses it [6].

Neuroblastoma (NB) is a heterogenic pediatric cancer with different outcomes, resulting in spontaneous regression with differentiation to benign tumors or rapid progression with a fatal outcome, causing 15% of cancer-related deaths in children [7]. Originating from neural crest-derived precursor cells, NB is characterized by the presence of different transcription factor proteins (c-, N-, and L-MYC), which are encoded by members of MYC proto-oncogene family [8].

The excessive expression of MYC proteins causes aggressive behavior, associated with altered survival/proliferation and drug-resistance in cancer cells [9]. Among the MYC family of oncoproteins, over-expression of MYCN is restricted in some cancers, including NB. In normal conditions, gene expression in neuroblasts is strictly controlled [10], while in culture conditions it becomes susceptible to changes that rapidly modify cell signaling and protein expression [11,12,13]. Thus, cultured NB cells can be used to investigate tumor behavior.

The over-expression of *MYCN* may be associated with increased cell adhesion to the extracellular matrix (ECM), which is considered as a starting event for cell regulation, including survival, proliferation, differentiation, and gene expression [14,15,16].

Among several cancer-related genomic alterations, *MYCN* gene amplification, leading to MYCN protein overexpression, has been found in about 20% to 25% of NB cases [17,18], and is associated with increasing disease severity, rapid progression and poor disease outcome [19]. Moreover, MYCN overexpression represents the main marker of poor prognosis, thereafter NB treatment changes on the basis of the MYCN status [20].

It is well known that hypoxic conditions are able to change tumor microenvironment that influences cell functions and differently modulates cell response. These latter, in turn, may be dependent on the activation of several transcription factors, including the hypoxia-inducible factor (HIF). Recently, we reported that CO_2_-induced hypoxic condition exerts cytotoxic effects on SH-SY5Y neuroblastoma cells by eliciting cell cycle arrest and mitochondrial apoptotic pathway, thereby improving the understanding of the possible clinical impact of CO_2_ pneumoperitoneum on NB behavior [21,22].

In this study, we aimed to investigate the role of TG2-L and TG2-S in NB cells cultured under hypoxic conditions (1% O_2_). In order to do this, we used two NB cell lines, namely *MYCN*-amplified IMR-32 cells and SH-SY5Y cells, that are non-*MYCN*-amplified [23] and are considered less invasive than IMR-32 cells. Both cell lines have been largely used in experimental studies as in vitro model to evaluate biochemical mechanisms involved in cancer modifications.

## 2. Results and Discussion

It has been reported that the outcome of NB treatment may be influenced by the hypoxic status, and intermittent hypoxia or repeated episodes of hypoxia followed by re-oxygenation are a common feature of NB [22,24]. Hypoxia may cause necrosis and apoptosis, cell cycle arrest and differentiation [25], but it is well known that in cancer cells a hypoxic environment may also stimulate tumor growth, invasion and metastasis, throughout the activation of a lot of transcription factors [26,27,28]. In particular, cell response to hypoxia is mainly mediated by the transcription factor HIF-1, that is able to modulate the expression of hundreds of genes involved in cell adaptation to low oxygen availability [29].

**Figure 1:**
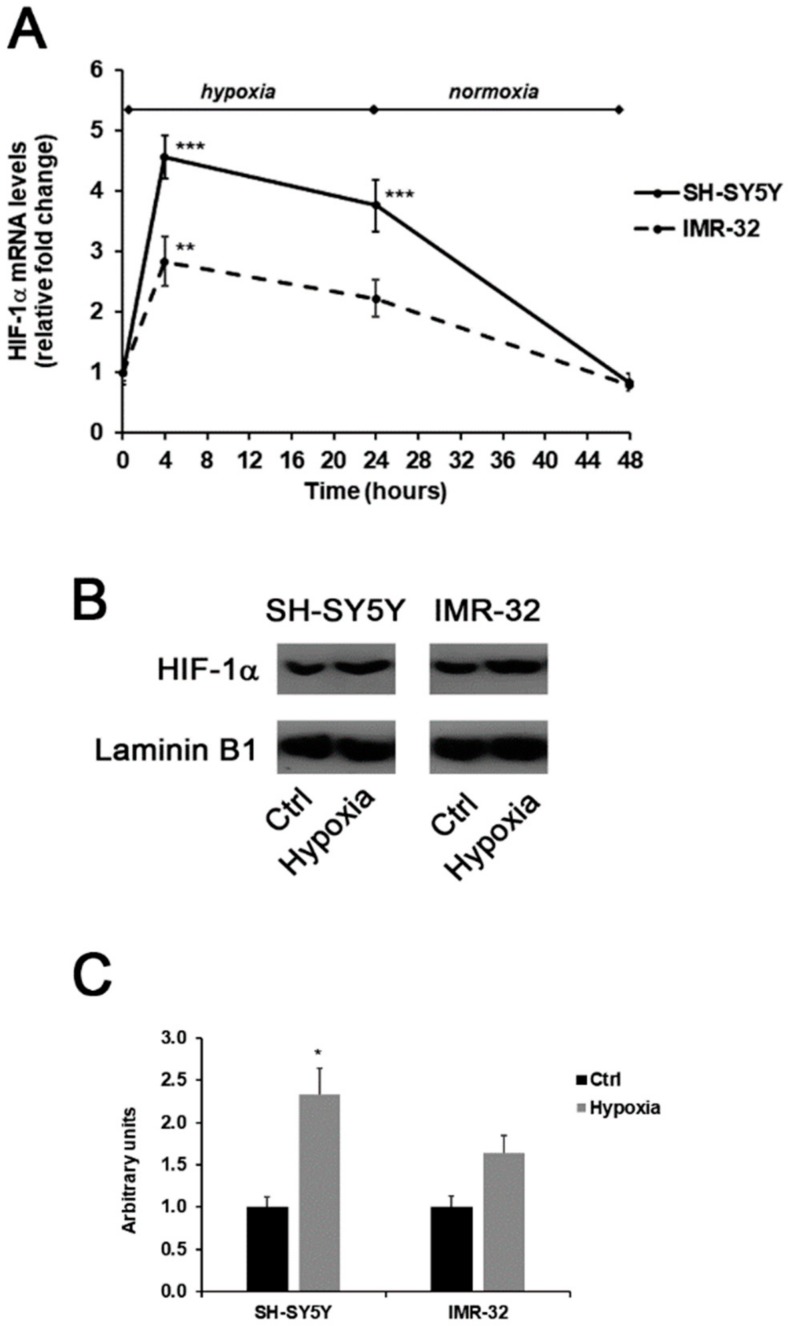
Effects of the exposure to hypoxia on the expression of HIF-1α in SH-SY5Y and IMR-32 NB cells. After incubation times, cells were harvested and used for the analysis of HIF-1α mRNA levels by Real-Time PCR (**A**); the nuclear content of HIF-1α protein in cells incubated for 4 h in hypoxic condition was assessed by Western blotting (**B**) and subsequent densitometric analysis (**C**). Data are expressed as mean ± SEM. *p < 0.05, **p < 0.01 and ***p < 0.001 significant differences in comparison to controls.

In this experimental study, the generation of a hypoxic environment induced the up-regulation of *HIF-1α* in both SH-SY5Y and IMR-32 cells. As shown in Figure 1A, hypoxia immediately induced a significant increase of about 4.5-folds in *HIF-1α* mRNA levels in SH-SY5Y cells in comparison to unexposed cells. Also, in IMR-32 cells the expression of *HIF-1α* increased after 4h of hypoxia, although at a less extent in comparison to SH-SY5Y cells. In both cell lines, HIF-1α mRNA levels returned to basal levels after 24 h of reoxygenation (Figure 1A). In addition, after 4 h of hypoxia the nuclear protein content of HIF-1α was increased in both cell lines (Figure 1B,C).

Although several results demonstrated the involvement of TG2 in the cross-talk between HIF activation and underlying mechanisms associated with proliferation, migration, and apoptosis [30,31], we also investigated the role of TG2 under hypoxic conditions in *MYCN*-amplified IMR-32 cells in comparison to the non-*MYCN*-amplified SH-SY5Y human NB cells.

It has been reported that an increase of TG2 expression confers a growth advantage to cancer cells, enabling them to survive in hypoxic conditions. TG2 overexpression seems to be associated with the proliferation and metastasis of various tumours, such as breast cancer, ovarian cancer, lung cancer and colorectal carcinoma [32,33,34,35,36]. Several reports proposed TG2 inhibition as a useful tool for therapeutic approaches. For example, it has been reported that the GK921 TG2 inhibitor, overlapping with the p53-binding site of TG2, was able to induce apoptosis in renal cell carcinoma [37]. Similarly, the inhibition of TG2 by the microRNA (miR)-214 was associated with a relevant suppression of viability, invasion and migration of colorectal carcinoma cells [36].

However, it has also been demonstrated that the induction of TG transamidating activity produces a reduction in tumor cell plasticity, migration and invasion [38]. It has been hypothesized that the different impact of TG2 on tumor behavior can be dependent on its enzymatic and non-enzymatic action in function of cell type, its intra- and extracellular localization, and substrate availability [3]. In addition, the numerous enzymatic activities and functions of TG2 may be attributed to alternative TG2 isoforms derived by alternative splicing of TG2 mRNA [39].

In the present study, we aimed to assess the occurrence of *TGM2* gene alternative splicing and the expression levels of TG2-L and TG2-S isoforms in NB cells, cultured in hypoxic and normal O_2_ conditions. Moreover, considering the involvement of *MYCN* in NB development mediated by TGM2 gene repression [40], we compared the expression levels of TG2 isoforms in *MYCN*-amplified IMR-32 cells and non-*MYCN*-amplified SH-SY5Y cells under hypoxia/reoxygenation conditions in order to assess if *TGM2* expression is influenced by a different *MYCN* amplification status.

**Figure 2:**
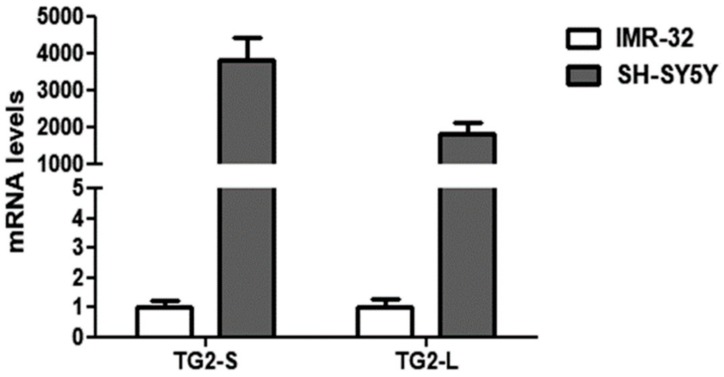
Comparison of basal transcript levels of the two TG2 isoforms, TG2-L and TG2-S, between SH-SY5Y and IMR-32 NB cells cultured in normal O_2_ conditions. The relative mRNA levels of TG2-L and TG2-S in SH-SY5Y are expressed as fold changes in comparison to IMR-32 cells. Data are expressed as mean ± SEM.

Our preliminary results demonstrated a remarkable down-regulation of both TG2 isoforms in the IMR-32 in comparison to the SH-SY5Y cells in normal O_2_ conditions (Figure 2).

These data are in agreement with the study of Liu et al. [40], demonstrating the reduction of *TGM2* mRNA transcripts in NB cells overexpressing *MYCN*. Given the well-known role of TG2 in NB cell differentiation [41], the low levels of TG2 could contribute to the aggressiveness of IMR-32. In this regard, it has been demonstrated that the induction of TG2 expression is necessary in promoting neurite outgrowth [42], and elevated levels of TG activity have been reported during axonal regeneration and during central nervous system development [43].

Previous observations demonstrated a high variability in the expression levels of TG2 isoforms in cancer cell lines and indicated that alternative splicing of TGM2 gene is an active process in tumor cells [39]. Moreover, it has been found that the TG2-S isoform, lacking the C-terminal GTP binding regulatory domain, which controls the response of TG2 to Ca^2+^ activation [44], played an opposite role to TG2-L in NB cells, favouring cell differentiation through its transamidation activity unlike TG2-L, which was active as a repressor of cell differentiation [6].

**Figure 3:**
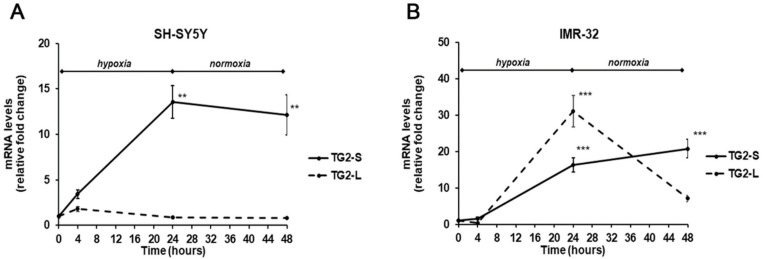
Differential expression of TG2-L and TG2-S transcripts in SH-SY5Y (**A**) and IMR-32 (**B**) NB cells after exposure to hypoxic conditions (1% O_2_) up to 24 h followed by reoxygenation for further 24 h. After incubation times, cells were harvested and the mRNA levels were evaluated by Real-Time PCR. Data are expressed as mean ± SEM. **p < 0.01 and ***p < 0.001 significant differences in comparison to controls.

In our experimental conditions, the exposure to hypoxia for 24 h induced a significant increase (13.5-folds, *p* < 0.01) of TG2-S mRNA levels in SH-SY5Y cells, that was maintained even after reoxygenation (12.1-folds, p<0.01) in comparison with unexposed cells. In the same cell line, no significant changes were observed in TG2-L transcripts following hypoxia (Figure 3A). In IMR-32 cells both isoforms of TG2 were significantly increased in hypoxic condition. In particular, the TG2-S increased after hypoxia and reoxygenation by 16.3-folds and 20.8-folds (*p* < 0.001), respectively. Similarly, the expression of TG2-L significantly increased after 24 h of hypoxia by 31-folds, and although decreased at the end of reoxygenation, remained about 7-fold higher than in unexposed cells (Figure 3B).

In the light of these results, it is possible to hypothesize that in IMR-32 NB cells hypoxia hinders the *MYCN*-mediated downregulation of *TGM2* gene as previously reported in cells grown in normal O_2_ conditions [40]. However, further experiments are necessary to sustain this hypothesis.

**Figure 4:**
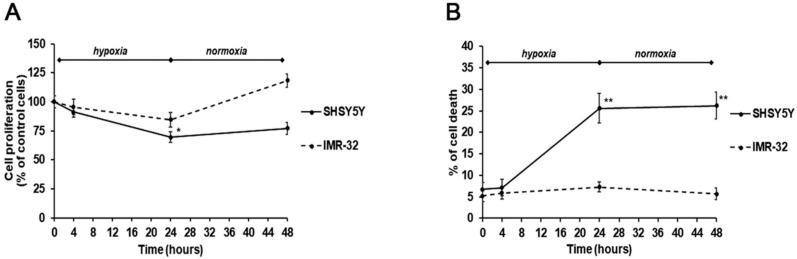
Cell proliferation (**A**) and cell death (**B**) assessed by MTT ((3-(4,5-methylthiazol-2-yl)-2,5-diphenyl-tetrazolium bromide)) and trypan blue exclusion assays in SH-SY5Y and IMR-32 NB cells unexposed and exposed to hypoxic conditions (1% O_2_) up to 24 h followed by reoxygenation for further 24 h. Results are expressed as percentages of the values detected in control cells. Each value is the mean ± SEM. *p < 0.05 and **p < 0.01 significant differences in comparison to unexposed cells.

Considering the distinct effects of the two TG2 protein isoforms exerted on NB growth, we analyzed cell proliferation and survival in hypoxic and normal O_2_ conditions, and after re-oxygenation. Notably, a different behavior of the two cell lines was observed. In particular, after 24-h hypoxia, a significant reduction of cell proliferation, concomitant with an increase of cell death, was observed in SH-SY5Y (Figure 4A,B). These effects were also persistent after reoxygenation for 24 h (Figure 4A,B). On the contrary, the IMR-32 cells proliferation and cell death were not significantly affected by hypoxic conditions and after re-oxygenation a slightly increase of cell proliferation was observed (Figure 4A,B).

These effects could be due to the different expression of the TG2 isoforms. In particular, it is possible to hypothesize that increased levels of TG2-L make IMR-32 cells more resistant than SH-SY5Y cells to the cytotoxic effect induced by hypoxia. The present observations contribute to explain the role of TG2 in NB development. It has been reported that transamidating activity is necessary for NB cell differentiation. In this regard, it is known that both TG2-L and TG2-S isoforms possess the transamidase catalytic site, whereas TG2-S lacks the Arg-580 residue that is critical for GTP binding and inhibition of enzyme activity. Further, it has been shown that TG2-L reduced NB cell differentiation, and TG2-L silencing in NB cells induced neuritic differentiation [6]. Thus, we can hypothesize that in IMR-32 cells the high expression levels of the TG2-L isoform, associated with the GTP binding and inhibition of its transamidating activity, could enhance cell survival under hypoxic conditions.

The moderate rise of cell proliferation after reoxygenation suggests an increase of IMR-32 aggressiveness, according to previous results [45], which indicated an aggressive hypoxic phenotype in NB cells persisting for 24 h or more, and making cells more prone to invasion and metastasis.

**Figure 5:**
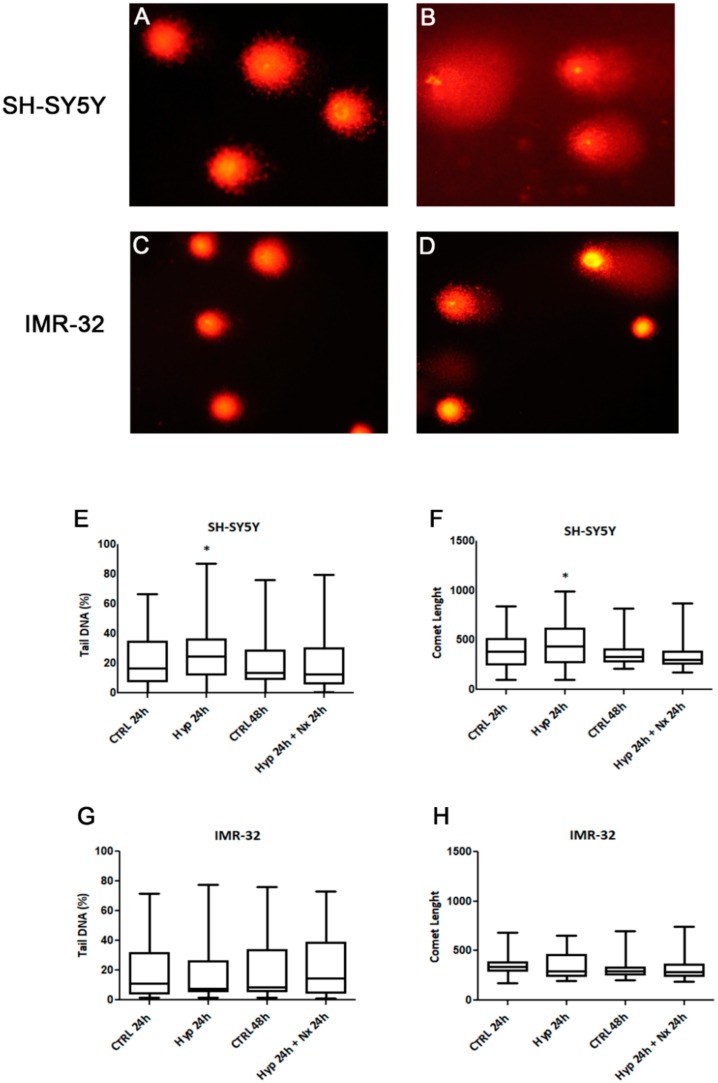
Comet assay pictures of DNA fragmentation in SH-SY5Y (**A**,**B**) and IMR-32 (**C**,**D**) NB cells exposed to normal O_2_ (**A**,**C**) and hypoxic (**B**,**D**) conditions for 24 h., and graphs of Comet parameters calculated in cell cultures exposed or not to hypoxia for 24 h followed by reoxygenation for further 24 h (**E**–**H**). Values are expressed as median, 25th and 75th percentiles and minimum and maximum. *p < 0.05 significant difference in comparison to control cells.

We also investigated if observed changes in cell proliferation and death could be related to hypoxia-induced DNA damage. It is recognized that the hypoxia induces genotoxicity though ROS formation and also by inhibiting the repair mechanisms of DNA [46]. The analysis by Comet assay showed a DNA damage accumulation after 24 h hypoxia treatment in SH-SY5Y cells compared to unexposed cells (*p* < 0.05, Figure 5A,B,E,F). However, no significant DNA damage was observed in the cells after reoxygenation for 24 h, as evidenced by analysis of tail DNA percentage and comet length (Figure 5 E,F). The DNA damage in SH-SY5Y was associated with a strong increase of TG2-S, but not TG2-L, and cell death. Indeed, in this context the lack of the GTP binding site in TG2-S could result in a high transamidating activity dependent also on the increase of intracellular calcium generated by hypoxic stress [47].

In IMR-32 cells we did not found differences in DNA damage parameters after hypoxia (Figure 5C,D), no changes in tail DNA percentage and comet length were evidenced in hypoxia exposed cells in comparison to unexposed cells (Figure 5G,H).

Cell response to hypoxia was associated with an increase of expression levels of both TG2 isoforms. Considering the well-known anti-apoptotic role of TG2-L, it is possible to hypothesize that in IMR-32 the activity of this isoform overrides the effects of TG2-S, making the cells more resistant to hypoxic conditions and also contributing to the gain in cell proliferation.

On the basis of these results, we can hypothesize that the expression of different TG2 isoforms observed in the two cell lines, bearing or not *MYCN* amplification, is associated with a different NB cell resistance to hypoxia-induced DNA damage. However, further experiments including TG2 silencing, are necessary to better clarify the role of TG2 isoforms in NB. Moreover, it is possible to hypothesize that *MYCN* amplification makes NB cells resistant to hypoxia-induced DNA damage. Indeed, it has been demonstrated that MYCN knockdown changed nucleosome organization and chromatin states resulting in DNA damage in NB [48].

Finally, we also assessed cell cycle progression through flow cytometry, as well as the mRNA and protein expression levels of cyclin B1 and D1 by Real-time PCR and Western Blotting, and the mRNA expression of tumor suppressor gene p16 by Real-time PCR.

**Figure 6:**
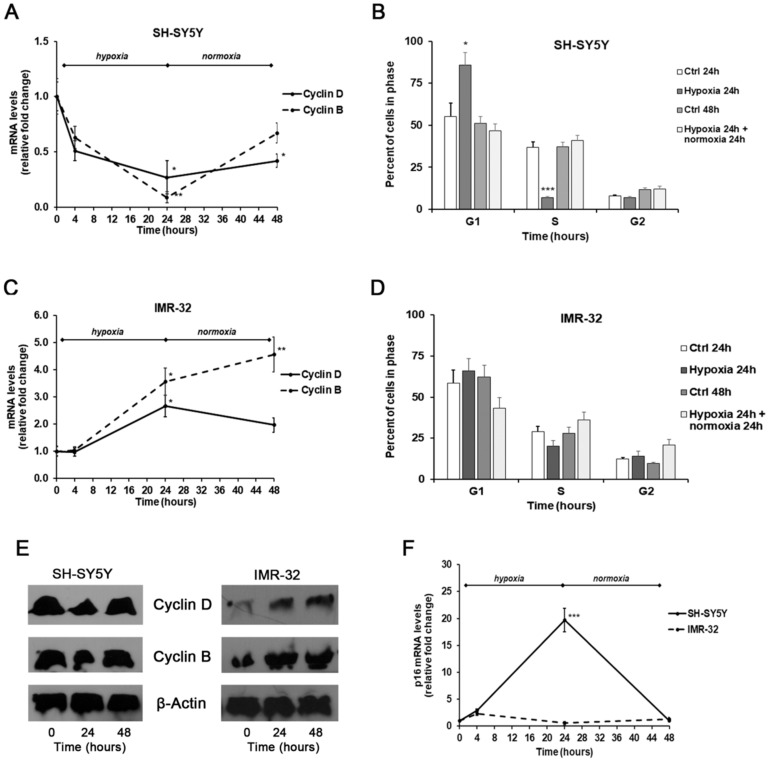
Effects of the exposure to hypoxia on cell–cycle progression. mRNA levels of cyclin D1, B1, and p16 in SH-SY5Y and IMR-32 NB cells were evaluated by Real Time-PCR (**A**,**C**,**F**). Protein levels of cyclin B1 and D1 were evaluated by Western Blotting (**E**). Cell-cycle progression in SH-SY5Y (**B**) and IMR-32 (**D**) NB cells was measured by FACS analysis. Histogram represents the percentages of cells in G1, S, and G2/M-phases. Data are expressed as mean ± SEM. *p < 0.05, **p < 0.01 and ***p < 0.01 significant differences in comparison to controls.

In SH-SY5Y exposed for 24 h to hypoxic conditions we found a remarkable decrease of both cyclin D1 and B1 levels compared with unexposed cells, that was associated with a considerable increase of p16 mRNA amounts. These events were reverted after 24 h of reoxygenation, although the levels of both cyclins remained below basal levels observed in unexposed cells (Figure 6A,E); instead, the p16 levels were similar to those found in unexposed cells (Figure 6F).

Under hypoxic conditions the SH-SY5Y cells were found predominantly in G1 phase (*p* < 0.05) and a smaller number in S phase (*p* < 0.001) in comparison to unexposed cells, indicating the entry of cells in a quiescent phase [49]. Instead, reoxygenated cells were distributed similarly to control cells (Figure 6B).

In comparison to SH-SY5Y, in IMR-32 cells the hypoxia conditions induced different effects on cell cycle markers. After exposure to hypoxia for 24 h, there was a significant increase of cyclin D1 expression (*p* < 0.05) that decreased in reoxygenated cells. In hypoxic cells we also observed a considerable increase of cyclin B1 expression that after reoxygenation increased by about 4.5-folds in comparison to cells grown in normal O_2_ conditions (Figure 6C,E). In IMR-32 no significant changes were observed in the mRNA levels of p16 both after hypoxia and reoxygenation incubations (Figure 6F). The cell cycle analysis did not show relevant changes in the number of cells in the different phases (Figure 6D). The up-regulation of cyclin D1 and B1 expression observed in IMR-32 could be responsible of the slight increase of cell proliferation.

Notably, it has been demonstrated that *MYCN* promotes the glycolysis and cell growth thus maintaining a proliferating phenotype [50]. Accordingly, in *MYCN*-amplified IMR-32, after hypoxia exposure we found an increase of cyclin D1 and B1 sustaining cell proliferation. On the contrary, in non-*MYCN*-amplified SH-SY5Y cells exposed to hypoxia we observed decreased cyclin D1 and increased p16 levels associated with a reduction of cell proliferation.

## 3. Materials and Methods

### 3.1. Materials

The human SH-SY5Y (CRL-2266) and IMR-32 NB cells were purchased from American Type Culture Collections (ATCC) (Rockville, MD, USA). RPMI, penicillin/streptomycin mixture, glutamine, phosphate buffered saline solution (PBS), mouse monoclonal antibodies for laminin B1, *HIF-1α*, cyclin B1, cyclin D1 and β-actin, horseradish peroxidase (HRP)-conjugated anti-mouse secondary antibody, X-ray film, developer and fixer, and other chemicals of analytical grade were from Sigma (Milan, Italy). Foetal bovine serum (FBS), TRIzol reagent, High-capacity cDNA archive kit, Sybr-green RT-PCR master mix, and Pierce ECL detection system were from Life Technologies (Milan, Italy). Primers were synthesized by Eurofins Genomics (Milan, Italy).

### 3.2. Cell Culture and Treatment

SH-SY5Y and IMR-32 NB cells were cultured in RPMI-1640 medium containing 10% (*v/v*) heat-inactivated FBS, L-glutamine (2 mM), 100 units/mL penicillin, and 100 mg/mL streptomycin, and maintained at 37°C in a humidified incubator with 5% CO_2_ and 95% air.

Sub-confluent cells were exposed to hypoxia (1% O_2_, 5% CO_2_, and 94% N_2_) for 24 h at 37 °C. A subset of cells was harvested after both 4 h and 24 h of hypoxia, while the other cells were incubated again under normal O_2_ conditions (95% air/5% CO_2_) for 24 h. Control cultures were maintained in a standard cell incubator (95% air/5% CO_2_) at 37 °C for the same times of incubation and either harvested before treatment begins (0 h), or after 4 h, 24 h, and 48 h.

### 3.3. Proliferation Assay and Cytotoxicity Study

To assess the effects of CO_2_ exposure on cell proliferation, an MTT (3-(4,5-methylthiazol-2-yl)-2,5-diphenyl-tetrazolium bromide) reduction assay was performed. At the end of the incubation periods, control and hypoxia exposed cells, seeded at a density of 2.5 × 10^4^ cell/well in 96-well culture plates, were incubated with fresh medium containing MTT (0.5 mg/mL) at 37 °C for 4 h. Then, insoluble formazan crystals were dissolved in 100 μL of acidic isopropanol (0.04 M HCl in absolute isopropanol) at 37 °C for 1 h. The optical density in each well was determined at 570 nm using a microplate reader (Tecan Italia, Cologno Monzese, Italy). Results are expressed as percentage of unexposed cells. All experiments were performed in triplicate.

Cytotoxic effects of CO_2_ exposure were assessed by the trypan blue dye exclusion test, according to manufactured instructions.

### 3.4. Analysis of Gene Expression by Real-Time PCR

Total RNA was isolated using TRIzol reagent, and then two micrograms of RNA were reverse transcribed with High-Capacity cDNA Archive kit according to the manufacturer’s instructions.

*HIF-1α*, TG2-L, TG2-S, Cyclin D1, Cyclin B1, *p16* mRNA levels were analyzed by SYBR green Real-Time PCR. β-actin was used as endogenous control. Quantitative PCR reactions were set up in duplicate in a 96-well plate and were carried out in 20 µL reactions containing 1× SYBR green PCR Mastermix, 0.1 µM specific primers, and 25 ng RNA converted into cDNA. RT-PCR was performed in a 7900HT Fast Real-Time PCR System (Life Technologies, Milan, Italy) with the following profile: one cycle at 95 °C for 10 min, followed by 40 cycles at 95 °C for 15 s and 60 °C for 1 min. A standard dissociation stage was added to assess primer specificity. The primer sequences used for Real-Time PCR are reported in Table 1 [39,51].

Data were collected and analyzed using SDS 2.3 and RQ Manager 1.2 software (Applied Biosystems, Foster City, CA) using the 2^−ΔΔCT^ relative quantification method. Values are presented as fold changes relative to unexposed cells.

### 3.5. Western Blotting

Cytoplasmic and nuclear extracts from exposed and unexposed cells were prepared as previously described [52]. The presence of HIF-1α in cell nuclear extracts and the cytoplasmic cyclins B1 and D1 were evaluated by Western blot analysis.

Protein concentration was evaluated by the Bradford method and 20 µg of proteins were loaded on a 10% denaturing SDS-polyacrylamide gel, and transferred to nitrocellulose membranes. After protein transfer, the membranes were blocked with 5% non-fat dry milk at room temperature for one hour. Detection of specific proteins was done by probing membranes with specific mouse monoclonal antibodies (diluted 1:1000 in TBS-T) overnight at 4 °C, followed by incubation with horseradish peroxidase-conjugated anti-mouse secondary antibody (diluted 1:3000 in TBS-T) for 2 h at room temperature. Immunoblots were developed with ECL Plus chemiluminescent detection system kit, using Kodak film. The bands were scanned and quantified by densitometric analysis with ImageJ 1.45 software (NIH, Bethesda, MD, USA) (http://rsb.info.nih.gov/ij/) using laminin B1 or β-actin for normalization.

### 3.6. Comet Assay

DNA damage was evaluated using a Single-cell gel electrophoresis (SCGE) technique, as previously reported by Montalto et al. [21]. Samples were run in duplicate, and images of 50 cells per slide for a total of 100 cells per sample were randomly acquired and analyzed using CASP software (Comet assay software project). The considered parameters were the percentage of DNA in the tail and the comet length.

### 3.7. Cell Cycle Analysis by Flow Cytometry

The distribution of cells in the cell-cycle phases was determined by flow cytometric analysis of the DNA content. After stimulation, SH-SY5Y and IMR-32 cells, seeded at a density of 2.5 × 10^5^ cells/mL in 12-well plates, were collected by non-enzymatic cell dissociation solution and centrifuged for 5 min at 300g. Then, the cells were fixed in cold 70 % ethanol at 4 °C for 2 h, resuspended in 500 µL of staining solution (50 µg/mL propidium iodide and 500 µg/mL RNase A in PBS) for 30 min at 37 °C and analyzed using a flow cytometer (Dako Galaxy Cytomation, Glostrup, Denmark).

### 3.8. Statistical Analysis

All experiments were repeated at least three times and each experiment was performed in triplicate. All values, except COMET parameters, are expressed as mean ± SEM. Statistical analysis was carried out using the *t*-test and One-Way ANOVA followed by Bonferroni post-hoc test. Since the Comet parameters showed a non-normal distribution, median and 25th and 75th percentiles were used to describe the observed data and the analysis of independent variables was performed by the Kruskal–Wallis test. *p*-values less than 0.05 were considered significant.

## 4. Conclusions

Our results give evidence for an opposite response to hypoxia conditions of NB cells carrying a different *MYCN* amplification status and expressing different levels of TG2 isoforms. Further experiments are needed to characterize the regulatory mechanisms underlying the effects of *MYCN* status on the differential expression of TG2 isoforms in NB cells in hypoxic conditions. A deep understanding of this issue may be helpful to clarify whether surgical interventions based on CO_2_-induced pneumoperitoneum can negatively influence the treatment outcome, and whether TG2 isoforms may represent new markers for risk stratification of NB and/or new therapeutic targets.

## Figures and Tables

**Table 1:** Primer sequences used for Real-Time PCR.

Gene	Forward	Reverse
*Cyclin B1*	5′-ctgctgcctggtgaagag-3′	5′-cgcctgccatgttgatct-3′
*Cyclin D1*	5′-tattgcgctgctaccgttga-3′	5′-ccaatagcagcaaacaatgtgaaa-3′
*HIF-1α*	5′-cgttccttcgatcagttgtc-3′	5′-tcagtggtggcagtggtagt-3′
*p16*	5′-accagaggcagtaaccat-3′	5′-gtaggaccttcggtgact-3′
*TG2-L*	5′-ccttacggagtccaacctca-3′	5′-ccgtcttctgctcctcagtc-3′
*TG2-S*	5′-accgctgaggagtacgtctg-3′	5′-tcaacaaatgctccaggaa-3′
*β-Actin*	5′-ttgttacaggaagtcccttgcc-3′	5′-atgctatcacctcccctgtgtg-3′
